# Al_13_Fe_4_-Al Composites with Nanocrystalline Matrix Manufactured by Hot-Pressing of Milled Powders

**DOI:** 10.3390/ma15124241

**Published:** 2022-06-15

**Authors:** Marek Krasnowski, Stanislaw Gierlotka, Dariusz Zasada

**Affiliations:** 1 Faculty of Materials Science and Engineering, Warsaw University of Technology, Woloska 141, 02-507 Warsaw, Poland; 2 Institute of High Pressure Physics, Polish Academy of Sciences, Sokolowska 29/37, 01-142 Warsaw, Poland; xray@unipress.waw.pl; 3 Faculty of Advanced Technologies and Chemistry, Military University of Technology, Kaliskiego 2, 00-908 Warsaw, Poland; dariusz.zasada@wat.edu.pl

**Keywords:** metallic composites, intermetallics, nanocrystalline materials, mechanical alloying, powder compaction

## Abstract

The paper describes composites with the matrix containing a nanocrystalline intermetallic Al_13_Fe_4_ phase and microcrystalline aluminium. Mechanically alloyed Al_80_Fe_20_ powder, containing a metastable nanocrystalline Al_5_Fe_2_ phase, was mixed with 20, 30, and 40 vol.% of Al powder and consolidated at 750 °C under the pressure of 7.7 GPa. During the consolidation, the metastable Al_5_Fe_2_ phase transformed into a nanocrystalline Al_13_Fe_4_ phase. In the bulk samples, Al_13_Fe_4_ areas were wrapped around by networking Al regions. The hardness of the Al_13_Fe_4_-Al composites was in the range of 4.52–5.50 GPa. The compressive strength of the Al_13_Fe_4_-30%Al and Al_13_Fe_4_-40%Al composites was 805 and 812 MPa, respectively, and it was considerably higher than that of the Al_13_Fe_4_-20%Al composite (538 MPa), which failed in the elastic region. The Al_13_Fe_4_-30%Al and Al_13_Fe_4_-40%Al composites, in contrast, showed some plasticity: namely, 1.5% and 9.1%, respectively. The density of the produced composites is in the range of 3.27–3.48 g/cm^3^ and decreases with the increase in the Al content.

## 1. Introduction

Aluminium-based alloys are characterised by low density and good corrosion resistance. Among aluminium alloys, attention is attracted by, i.a., intermetallic phases of the Fe-Al system [1]. They have advantageous properties: a high specific strength and stiffness, good strength at intermediate temperatures, and excellent corrosion resistance at elevated temperatures [1]. Properties such as hardness and strength can be improved when iron aluminides have a nanocrystalline structure [2,3]. A well-known method of producing nanocrystalline materials is mechanical alloying (MA), followed by the consolidation of milled powders [4]. However, most consolidation techniques do not allow for obtaining of truly nanocrystalline samples that are large in size and free from artefactual defects formed during consolidation [5]. It was observed that such defects are a frequent cause of a lack of plasticity in consolidated samples [6]. These defects are often micropores between weakly bound powder particles, and they cause bulk nanocrystalline materials to crack at stresses lower than the yield strength. Therefore, the technological processes of bulk nanocrystalline materials can be treated as critical factors influencing their low ductility, and the key to its improvement is the consolidation of the material free of artefact-like defects [6].

High-temperature consolidation of mechanically alloyed powders, with a nanocrystalline structure preserved, is a task that is difficult to perform. The application of high temperatures, which is required to achieve good quality consolidation, can lead to excessive grain growth. As we previously demonstrated, application of high pressure during consolidation inhibits grain growth and maintains the nanocrystalline structure of the pressed powder [7,8].

It was reported that the presence of larger grains in a nanocrystalline matrix (bimodal grain distribution) can improve a material’s ductility in comparison with samples containing only nanometric grains [9]. A grain size distribution containing a fraction of grains large enough to maintain dislocation activity enables strain hardening, and thus favours an increase in ductility [6]. This prompted us to modify the microstructure of the consolidated powders to improve the ductility of the bulk materials [10,11]. We, therefore, produced the composite materials comprising a nanocrystalline intermetallic as a matrix and aluminium as a binder [10,11]. Ductile aluminium was to be distributed in the form of a “network” between hard nanocrystalline particles. The expected effect of the presence of a soft aluminium “network” was to improve the quality of bonding nanocrystalline particles into bulk material and, being a ductile component of the composite, to improve the plasticity of material. Micrometric aluminium grains contribute to the plastic deformation, and they may release stress concentrations and, hence, retard crack initiation and propagation [12]. The introduction of aluminium lowered the final density of the composites but also resulted in a decrease in the material hardness. Powders with nanocrystalline structure intended to form the matrix were produced by mechanical alloying [10,11]. They were then mixed with Al powder and subjected to short-term low-energy milling, which ensured even distribution of Al in the powder material. Mixtures prepared in such a way were consolidated into bulk form [10,11].

There are very few publications describing bulk materials containing ultrafine and larger grains, obtained by mixing nanocrystalline and microcrystalline powders, as well as the subsequent consolidation [9,12]. Apart from those reported by our group [10,11,13], no works related to nanocrystalline powders mixed with microcrystalline aluminium were found. It is, therefore, worth noting that the nanocrystalline phase-Al composites, as well as their technology, are innovative.

Recently, we prepared Al_3_Ni_2_-Al [10] and Al_5_Fe_2_-Al [11] composites with a nanocrystalline intermetallic matrix and various content of microcrystalline Al. The increase in composites’ plasticity with the increase in Al content was observed in both cases. In the present work, composites with the matrix containing a nanocrystalline intermetallic Al_13_Fe_4_ phase and microcrystalline Al are being reported. The Al_13_Fe_4_ intermetallic phase has the density lower than the Al_3_Ni_2_ or Al_5_Fe_2_ intermetallic phases, and hence, the composites produced in this work will have a lower density than those reported previously. Based on the results of the previous studies [14], the alloy with Al_80_Fe_20_ stoichiometry was selected for the production of Al_13_Fe_4_ phase.

## 2. Materials and Methods

Al_80_Fe_20_ (at.%) elemental powders mixture was mechanically alloyed under argon atmosphere in a SPEX 8000 D shaker ball mill (SPEX^®^ SamplePrep, Metuchen, NJ, USA). The starting powders were ABCR (ABCR GmbH & Co. KG, Karlsruhe, Germany) products: Al (99.7% purity, 325 mesh) and Fe (99.9% purity, 200 mesh). The ball-to-powder ratio was about 10:1. The total milling time was 35 h.

The produced Al_80_Fe_20_ alloy powder was blended with 20, 30, and 40 vol.% of elemental Al powder and milled for 20 min with low intensity (ball-to-powder weight ratio 3:1) to ensure uniform mixture of the two components. Low-intensity milling was carried out under argon atmosphere in a SPEX 8000 D mill.

The Al_80_Fe_20_-Al powder mixtures were consolidated using a press (ASEA, Västerås, Sweden) equipped with a toroid-type high-pressure cell (Institute for High Pressure Physics of the Russian Academy of Sciences. Troitsk, Moscow Region, Russia). The sample was placed inside a graphite tube with 1 mm thick wall serving as a resistance heater. The heater was enclosed in a toroidal container made of lithographic limestone with a 25 mm outer diameter. The assembly was compressed uniaxially between two tungsten carbide anvils using a press. The anvils’ shape and the gasket’s material ensure that the pressure conditions are close to isostatic. The compaction processes were performed under a pressure of 7.7 GPa for 3 min. Pressure of 7.7 GPa was achieved with a compression force of 400 Tonnes. The pressing temperature was in the range of 600–800 °C. Pellets were loaded at the rate of 0.5 GPa/min prior to heating. The heating and cooling rate was 1000 °C/min. The consolidated samples were cylindrical in shape with a diameter of 5 mm and a height of about 4 mm.

The X-ray diffraction (XRD) investigations of the powders and consolidated samples were carried out in a Rigaku MiniFlex II X-ray diffractometer (Rigaku Corporation, Tokyo, Japan) operating with CuKα radiation.

A Hitachi S-3500N scanning electron microscope (SEM) (Hitachi high-tech corporation, Tokyo, Japan), equipped with an energy dispersive spectroscopy system (EDS), was used for examinations of the low-intensity milled powder mixtures and for the determination of chemical composition. Cross-section samples for SEM investigations were prepared by embedding the powder in a conducting resin and subsequently grinding and polishing it.

Particle size distribution measurements for the low-intensity milled powder mixtures were performed using a KμK mini3D (Kamika Instruments, Warszawa, Poland) analyser.

A Zeiss AXIOVERT 40 MAT light microscope (Carl Zeiss MicroImaging GmbH, Gottingen, Germany) and a Hitachi S-3500N SEM equipped with an EDS system were used for structural investigations and chemical analysis of the consolidated material. Bulk samples for these microscopy investigations were prepared by standard polishing techniques.

EBSD examination of a consolidated sample was performed using a Quanta 3D FEG high-resolution scanning electron microscope (FEI Company, Hillsboro, OR, USA). The sample for the EBSD investigation was prepared by grinding up to 4000 SiC paper, diamond suspension polishing and polishing with 0.1 μm SiO_2_ suspension.

A Zwick Roell hardness tester (ZwickRoell GmbH & Co. KG, Ulm, Germany) was employed for the measurements of Vickers hardness (HV1) of the compacted samples (average value of at least 25 indentations).

A Zwick Roell Z250 testing machine (ZwickRoell GmbH & Co. KG, Ulm, Germany) was used for uniaxial compression tests of bulk materials. Experiments were performed under displacement control at a strain rate of 10^−4^ s^−1^.

A Gibertini E154 (Gibertini Elettronica, Milano, Italy) balance equipped with a device for measuring the density of solids was employed for the determination of the bulk samples density (Archimedes method). The mass measurements performed allowed to calculate the open porosity of the consolidated samples.

## 3. Results and Discussion

**Figure 1:**
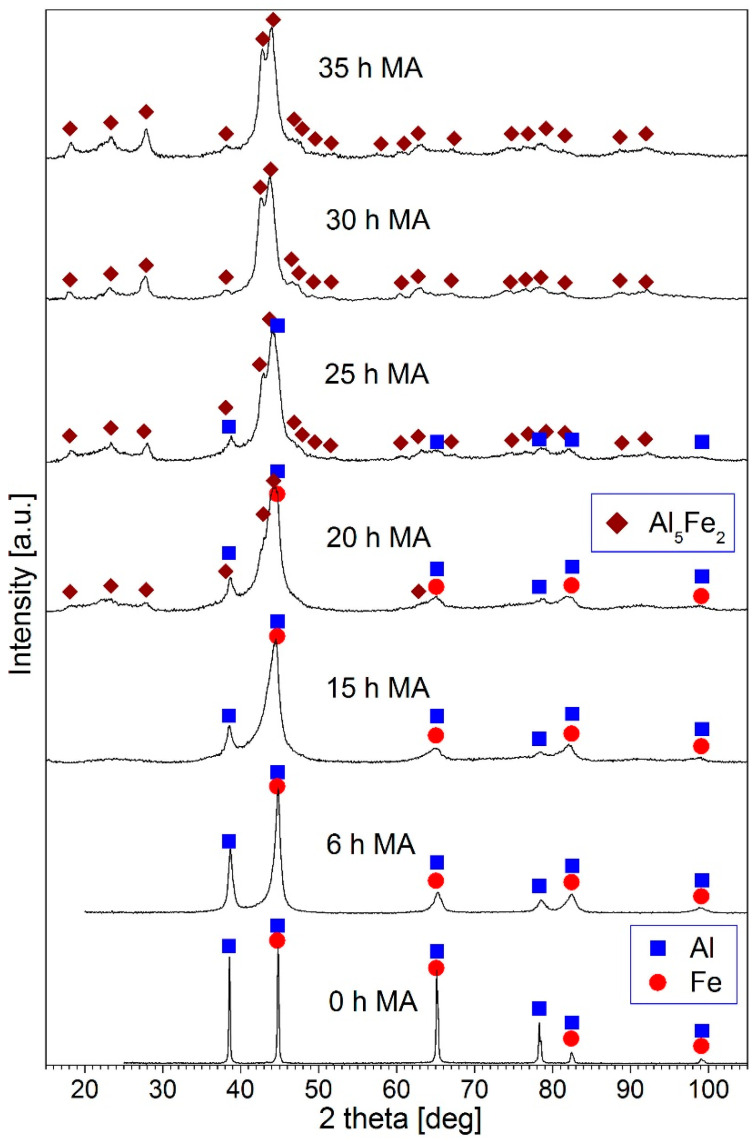
XRD patterns of the Al_80_Fe_20_ powder mixture milled for the times quoted.

Phase development and structural evolution in the Al_80_Fe_20_ powder mixture during mechanical alloying was studied by recoding of XRD patterns of the powders after various milling times (Figure 1). In the early stage of mechanical alloying (up to 15 h), a Fe(Al) solid solution was formed. This is evidenced by the gradual decrease in the intensity of the Al diffraction peaks compared to that of the Fe peaks and by the appearance of an asymmetry of the Fe diffraction profiles, which become wider on the low-angle side. Such features of the diffraction patterns accompanying the formation of a Fe(Al) solid solution were analysed in our earlier work [15]. In the diffraction pattern taken after 20 h of milling, a few broad peaks of low intensity appear. These peaks are assigned to Al_5_Fe_2_ intermetallic phase. In the pattern for 25 h-milled powder, more well-defined peaks of an Al_5_Fe_2_ phase are visible. In the XRD pattern after 30 h of milling, the peaks of Al and Fe(Al) are not present, which indicates that all the Al reacted with Fe, creating Al_5_Fe_2_ intermetallic, are at least partially ordered. Additionally, in the pattern of the 35 h-milled powders, only the peaks of Al_5_Fe_2_ phase are present. The observed phase evolution during mechanical alloying of the Al_80_Fe_20_ powder mixture is similar to that described earlier [14].

In the Fe-Al phase equilibrium diagram, the Al_5_Fe_2_ phase exists for the Al concentration of 70–72 at.% [16]. Thus, the produced Al_80_Fe_20_ alloy powder has a metastable phase composition. Mechanical alloying is a nonequilibrium process and the phase composition of its products most often differs from that expected from the phase equilibrium diagrams.

It was found that, during the heating of the mechanically alloyed Al_80_Fe_20_ powder in a calorimeter up to 720 °C, the Al_5_Fe_2_ phase transformed into the Al_13_Fe_4_ [14]. For the concentration of 80% Al, there is a two-phase Al_13_Fe_4_+Al area in the Fe-Al phase equilibrium diagram [16]. Hence, the heating in a calorimeter moved the phase composition of the powder towards equilibrium.

There are also reports on synthesis of an unknown Al-rich phase [17] or an amorphous phase [18,19] by mechanical alloying of an Al_80_Fe_20_ powder mixture. Even for the same composition of the initial powder mixture, products of differing phase composition and structure can be synthesised by mechanical alloying, depending on applied parameters, such as type of mill, milling intensity, etc. [4,14].

In the XRD patterns of the powders after milling time longer than 6 h, all the diffraction peaks are broad, which indicates nanometric crystallite size in the phases existing in the powders. 

**Figure 2:**
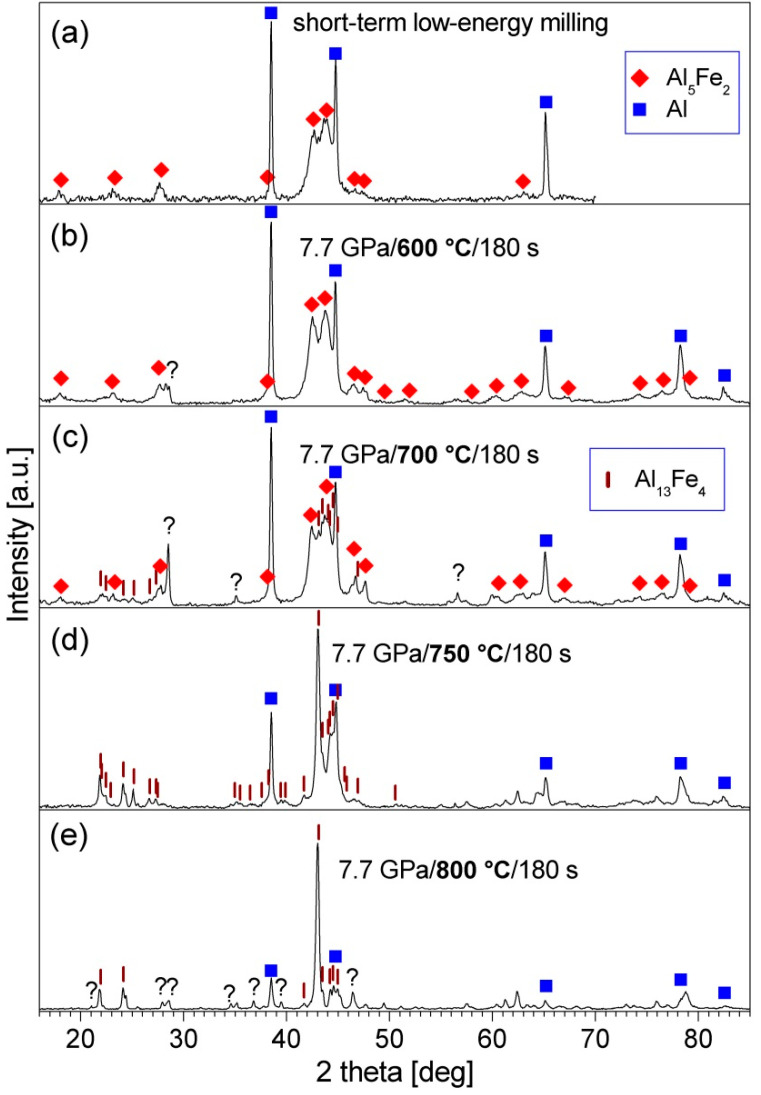
XRD patterns of the Al_80_Fe_20_-30%Al powder after: (**a**) short-term low-energy milling, (**b**) short-term low-energy milling and consolidation at 600 °C, (**c**) short-term low-energy milling and consolidation at 700 °C, (**d**) short-term low-energy milling and consolidation at 750 °C, and (**e**) short-term low-energy milling and consolidation at 800 °C.

**Figure 3:**
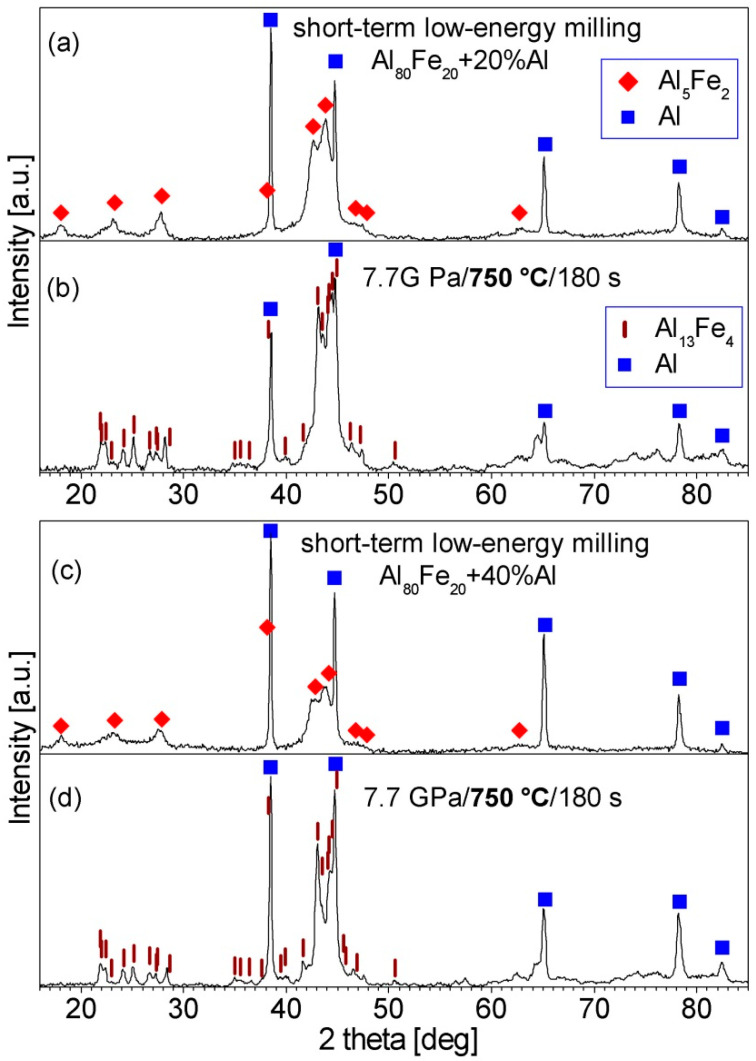
XRD patterns of the Al_80_Fe_20_-20%Al powder and Al_80_Fe_20_-40%Al powder after: (**a**,**c**) short-term low-energy milling, (**b**,**d**) short-term low-energy milling, and consolidation at 750 °C.

The produced Al_80_Fe_20_ alloy powder was blended with 20, 30, or 40 vol.% of Al and subjected to short-term low-energy milling. In the XRD patterns of the processed mixtures, only the diffraction peaks of Al_5_Fe_2_ and Al are visible (see Figure 2a and Figure 3a,c). Thus, the low-energy milling processes did not cause any reactions between the phases present in the powder blends.

**Figure 4:**
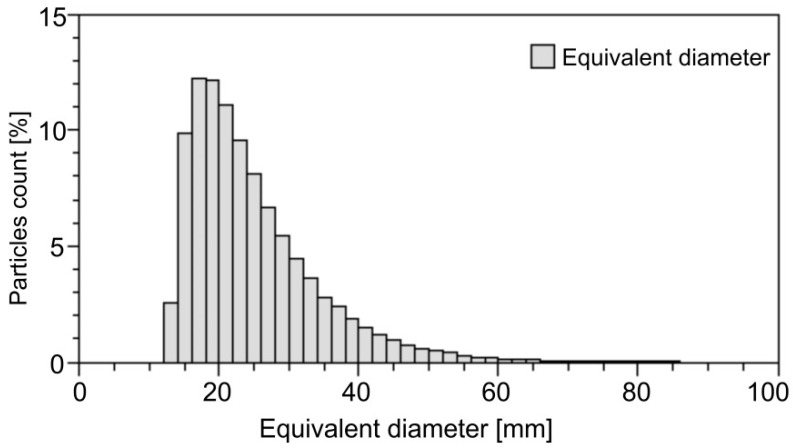
Particle size distribution for the short-term low-energy milled Al_80_Fe_20_-40%Al powder mixture.

To analyse particle size variation and particle shape, the powders after short-term low-energy milling were subjected to particle size distribution measurements and SEM investigations. Figure 4 illustrates particle size distribution for the short-term low-energy milled Al_80_Fe_20_-40%Al powder mixture as an example. The mean equivalent diameter of powder particles for this sample is 24.8 μm. For the other short-term low-energy milled powder mixtures, similar particle size distribution was found, and the mean equivalent diameter was 23.5 μm and 22.0 μm for the Al_80_Fe_20_-30%Al and Al_80_Fe_20_-20%Al powder mixtures, respectively.

**Figure 5:**
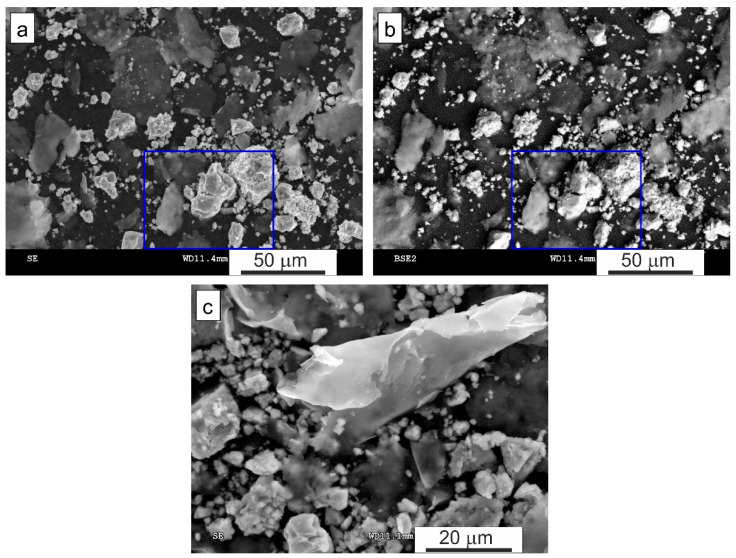
SEM images of the loose powder of the short-term low-energy milled Al_80_Fe_20_-40%Al mixture: (**a**) SE mode, (**b**) BSE mode (the rectangle marks the area selected for the EDS analysis, shown in Figure 6), and (**c**) SE mode (higher magnification).

Figure 5 shows SEM images of the loose powder of the short-term low-energy milled Al_80_Fe_20_-40%Al mixture as an example. In the powder mixture, two kinds of particles are visible: one, of almost spherical shape, visible as bright in BSE mode, and the other, with lamellar shape (Figure 5c), visible as grey in BSE mode (Figure 5b).

**Figure 6:**
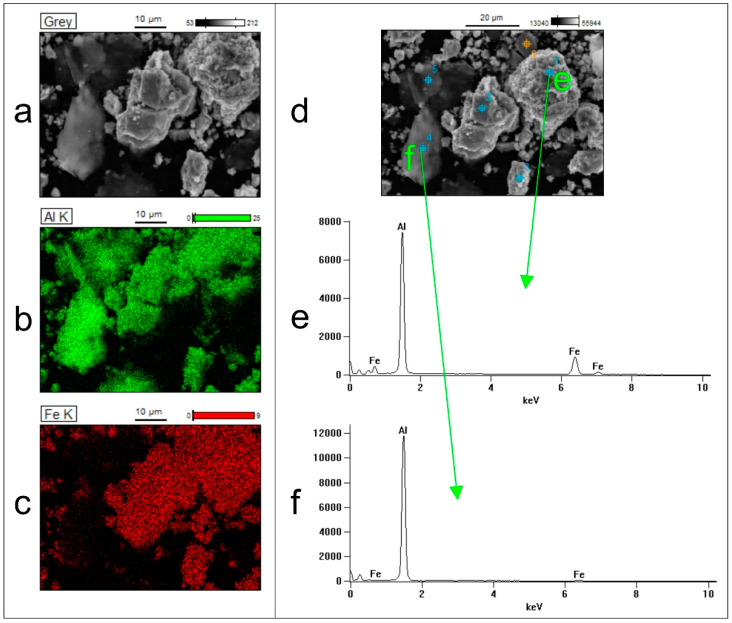
Results of EDS analysis of the area indicated in Figure 5: (**a**) SEM image, (**b**) EDS map with Al signal, (**c**) EDS map with Fe signal, (**d**) selected points for EDS analysis, (**e**) EDS spectrum taken in the point marked as “e” in (**d**), and (**f**) EDS spectrum taken in the point marked as “f” in (**d**).

EDS investigations revealed that the spherical particles are the Al_80_Fe_20_ alloy powder, while the lamellar particles are pure Al (Figure 6).

**Figure 7:**
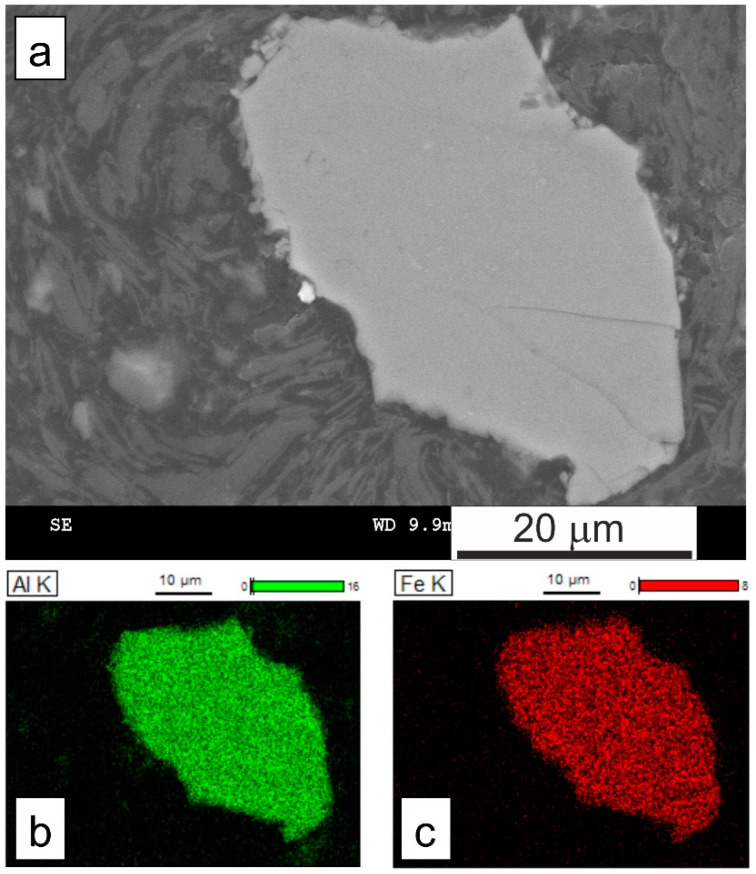
(**a**) SEM image of the cross-section of alloy powder particle in the short-term low-energy milled Al_80_Fe_20_-40%Al mixture: (**b**) EDS map for Al, (**c**) EDS map for Fe.

The alloy powder particles are mostly agglomerates, but there also exist some fine individual particles (Figure 5). Figure 7 depicts cross-section of an alloy powder particle. The EDS analysis of the alloy powder particles cross-sections demonstrated that the particles are chemically homogeneous, with an average composition of 78.5% Al and 21.5% Fe, as well as a composition fluctuation of 1%. For this composition, there is also a two-phase Al_13_Fe_4_+Al area in the Fe-Al phase equilibrium diagram [16]. Departure from the initial composition often occurs during mechanical alloying when one of the ground metals is much more plastic than the other and may stick to the grinding media (balls or container) a bit.

The prepared Al_80_Fe_20_-Al powders were consolidated by hot-pressing. The purpose was to produce a composite built of a nanocrystalline matrix, which is an Al_13_Fe_4_ phase (closer to the equilibrium state than an Al_5_Fe_2_ one for the Al_80_Fe_20_ composition) and an Al network that wraps around the matrix areas.

The use of high temperature during the consolidation of powders facilitates obtaining high quality compaction. However, in the case of multiphase powders, the application of high temperature can result in a reaction between phases. We reported this in the case of two-phase powders containing Al [11,20,21]. Therefore, the appropriate temperature must be used: high but not high enough to cause uncontrolled reactions. To select the pressing temperature, the Al_80_Fe_20_-30%Al powder was consolidated at 600, 700, 750, and 800 °C. The melting point of Al under atmospheric pressure is 660.3 °C, and increases with increasing pressure up to about 1180 °C at 7.7 GPa [22]. Thus, under the pressure applied in our work, we did not expect the melting of Al during consolidation at 700, 750, and 800 °C.

The XRD patterns of the Al_80_Fe_20_-30%Al powder before and after consolidation are shown in Figure 2. XRD investigations revealed that: (i) after consolidation at 600 and 700 °C, besides Al, the Al_5_Fe_2_ phase is still present in the material; (ii) during consolidation at 750 °C the Al_5_Fe_2_ phase transformed into the Al_13_Fe_4_ phase; (iii) during consolidation at 800 °C Al_13_Fe_4_ phase and unidentified phases formed. Based on these results, the temperature of 750 °C was chosen for the hot-pressing of the two other powders. In the XRD patterns of both consolidated powders, the diffraction peaks of the Al_13_Fe_4_ phase and Al are present (Figure 3b,d). Overlapping of the peaks of the Al_13_Fe_4_ phase made it impossible to reliably estimate the crystallite size of this phase, but the width of the peaks indicates the presence of a nanocrystalline structure in this phase.

**Figure 8:**
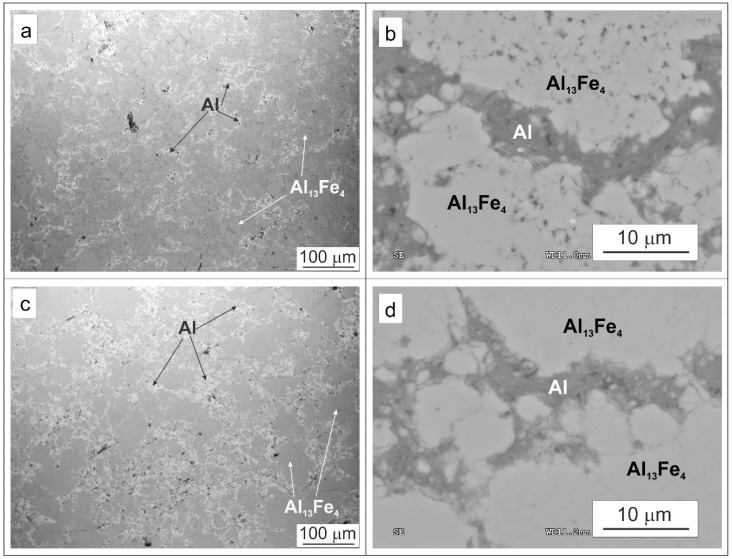
Micrographs of the Al_80_Fe_20_-20%Al bulk composite: (**a**) LM, (**b**) SEM, and micrographs of the Al_80_Fe_20_-40%Al composite: (**c**) LM, (**d**) SEM.

**Figure 9:**
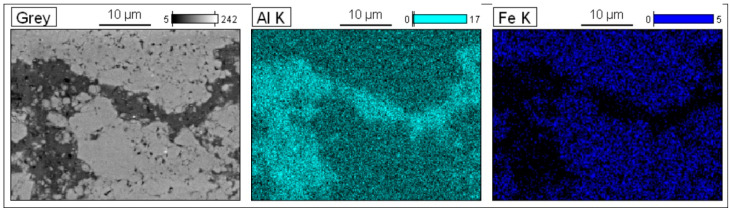
EDS maps of the Al_80_Fe_20_-20%Al bulk composite.

Light microscopy (LM) and SEM methods were used to observe the microstructure of the bulk samples and check the quality of consolidation. Examples of microstructure and EDS maps of the consolidated samples are shown in Figure 8 and Figure 9, respectively. The microstructure consists of matrix areas (dark in LM, bright in SEM), wrapped around by networking regions (bright in LM, dark in SEM). LM micrographs show that the share of the wrapping networking regions is larger in the samples with higher content of Al. Examples of EDS maps of the Al_13_Fe_4_–20%Al sample (Figure 9) show that the matrix areas contain Al and Fe, while in the wrapping regions, only Al is present. Microscopy observation, coupled with the XRD and EDS results, indicates that the networking regions are Al and the other areas make up the Al_13_Fe_4_ matrix. Thus, the bulk samples have Al_13_Fe_4_-Al composite microstructure. Similar microstructure, i.e., Al wrapping networking regions between regions of the nanocrystalline matrix, was obtained in the case of Al_3_Ni_2_-Al [10], Al_5_Fe_2_-Al [11], and TiC-Al [13] composites produced in a similar manner as the ones in this work.

Microscopy examination of the bulk samples showed that their surface is smooth, without pores, cracks, or voids.

**Figure 10:**
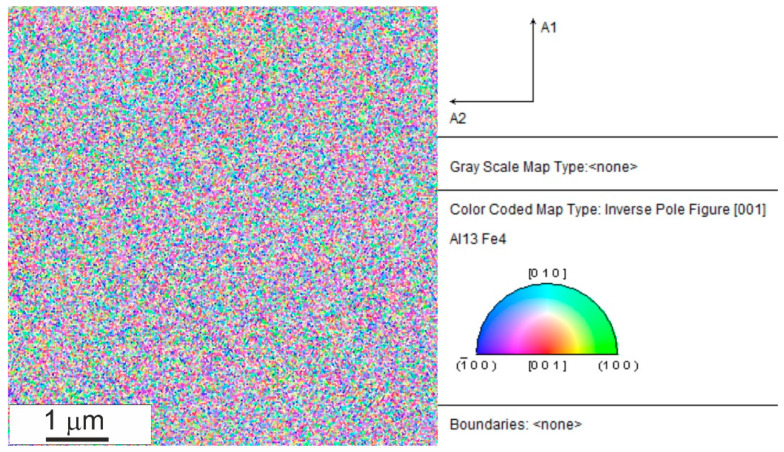
The EBSD inverse pole figure map of the matrix in the Al_80_Fe_20_-40%Al bulk sample.

The results of the XRD examinations of the consolidated samples, presented above, indicated the presence of a nanocrystalline structure in the Al_13_Fe_4_ phase being the composites matrix. To confirm a nanocrystallinity of the Al_13_Fe_4_ phase, the Al_80_Fe_20_-40%Al bulk sample was investigated in SEM by the EBSD method. Figure 10 presents the EBSD map of an area of the matrix in the bulk sample. As it can be seen, the crystallite size of the Al_13_Fe_4_ phase is below 100 nm. Thus, the produced bulk composites have nanocrystalline matrix. The preservation of the nanocrystalline structure of the intermetallic phase, during consolidation at high temperatures, is due to the use of high pressing pressure, as we have shown previously [7,8]. Since diffusion is involved in grain growth, the influence of pressure apparently comes from the fact that the diffusion coefficient decreases with the increase in pressure, usually by a factor ranging from 2 to 10 for a pressure of 1 GPa [23]. Hence, the use of high pressure during consolidation can reduce the mobility of grain boundaries at high temperature.

The produced composites were also characterised by compression tests, as well as hardness, density, and open porosity measurements. The hardness, compressive strength, plastic strain, and density values are given in Table 1.

**Figure 11:**
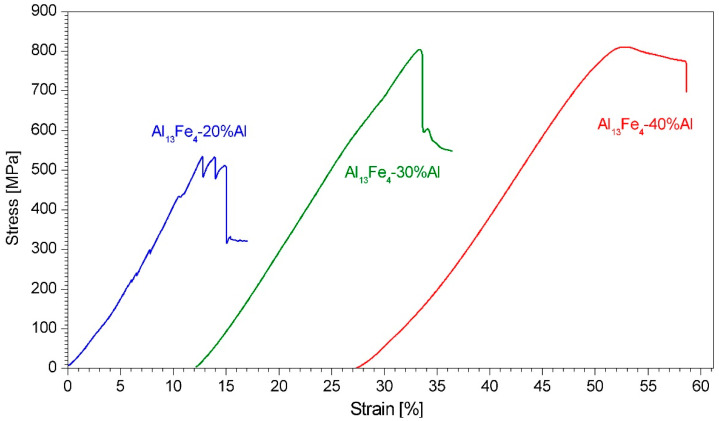
Compressive stress–strain curves of the consolidated Al-Al_13_Fe_4_ composites. The curves are shifted relative to each other for clarity.

The hardness of the composites decreases with the increase in the networking Al regions content. However, the compressive strength of the Al_13_Fe_4_-30%Al and Al_13_Fe_4_-40%Al composites is considerably higher than that of the Al_13_Fe_4_-20%Al one. This is because, during the compression test, the Al_13_Fe_4_-20%Al sample fractured catastrophically in the elastic region, crumbling into fine pieces. This is probably due to the insufficient bonding of the powder particles during the consolidation process. The Al_13_Fe_4_-30%Al and Al_13_Fe_4_-40%Al composites, in contrast, showed some plasticity, and, after failure, remained whole. However, the composite with 30% of Al, unlike that with 40% of Al, fractured shortly after yield. Thus, the presence of more than 20% of the networking Al in the Al_13_Fe_4_-Al composites improved the interparticle bonding and the ductility of the material. In addition, micrometric Al grains participate in the plastic deformation, and they can also help to release stress concentrations and, thus, retard crack initiation and propagation [12]. The compressive stress–strain curves are shown in Figure 11.

For the in-situ Al-Al_13_Fe_4_ composite, prepared by mechanical alloying of Al_90_Fe_10_ powder mixture followed by spark plasma sintering, the microhardness HV0.3 of 1.97 GPa and the compressive strength of 1130.1 MPa (but without any plasticity of material) were reported [24]. No other information concerning Al_13_Fe_4_-Al composites was found in the literature.

The density of the produced composites decreases with the increase in the Al content. The open porosity in all the produced composites does not exceed 0.15%. The specific compressive strength of the composites with 40% and 30% of Al is 248 kNm/kg and 235 kNm/kg, respectively, and is higher than that of Al_5_Fe_2_-Al (185-190 kNm/kg [11]) and Al3Ni2-Al (146-207 kNm/kg [10]) composites.

## 4. Conclusions

Mechanically alloyed Al_80_Fe_20_ powder containing a metastable nanocrystalline Al_5_Fe_2_ phase was used as a matrix for the composites. Al_80_Fe_20_-X vol.% Al (X = 20, 30, and 40) powder mixtures were consolidated by hot-pressing. Hot-pressing tests have shown that the temperature of 750 °C is suitable for the consolidation, during which only the transformation of the Al_5_Fe_2_ phase into the nanocrystalline Al_13_Fe_4_ phase occurs, without other reactions. The microstructure of the bulk samples consisted of Al_13_Fe_4_ intermetallic matrix areas wrapped around by networking Al regions. With the increase in Al content, the hardness of the composites decreased, ranging from 4.52 to 5.50 GPa. The Al_13_Fe_4_-20%Al composite failed in the elastic region, while the composites with 30% and 40% of Al showed some plasticity: namely, 1.5% and 9.1%, respectively. The presence of more than 20% of Al in the Al_13_Fe_4_-Al composites improved their ductility. The compressive strength of the composites containing 30 and 40% of Al was 805 and 812 MPa, respectively, and it was considerably higher than that of the Al_13_Fe_4_-20%Al composite (538 MPa). The low-density materials produced have a relatively high hardness.

## Figures and Tables

**Table 1:** Values of hardness (HV1), ultimate compressive stress (σ_UCS_), plastic strain (ε_p_), and density (ρ) of the produced composites.

Material	HV1 [GPa]	σ_UCS_ [MPa]	ε_p_ [%]	ρ [g/cm^3^]
Al_13_Fe_4_-20%Al	5.50	538	-	3.48
Al_13_Fe_4_-30%Al	5.09	805	1.5	3.42
Al_13_Fe_4_-40%Al	4.52	812	9.1	3.27

## Data Availability

Data sharing not applicable.
